# Global and national burden and trends of mortality and disability-adjusted life years for silicosis, from 1990 to 2019: results from the Global Burden of Disease study 2019

**DOI:** 10.1186/s12890-022-02040-9

**Published:** 2022-06-21

**Authors:** Shimin Chen, Miao Liu, Fei Xie

**Affiliations:** 1 Institute of Geriatrics, Second Medical Center, Beijing Key Laboratory of Aging and Geriatrics, National Clinical Research Center for Geriatric Disease, Chinese PLA General Hospital, Chinese PLA Medical School, Beijing, China; 2 Graduate School, Chinese PLA General Hospital, Chinese PLA Medical School, Beijing, China; 3 College of Pulmonary and Critical Care Medicine, Chinese PLA General Hospital, Beijing, China

**Keywords:** Silicosis, Global burden of disease, Age-standardized rate, Sociodemographic index, Joinpoint regression, Spatiotemporal trends

## Abstract

**Background:**

Silicosis, as an important type of pneumoconiosis, leads to progressive and irreversible conditions from the beginning of inflammation and fibrosis. However, the data on the global burden of silicosis and long-term trends were limited.

**Methods:**

Derived from the Global Burden of Disease study 2019 (online publicly available: Global Health Data Exchange), data on both crude and age-standardized rates (ASR) per 100,00 people of mortality and disability-adjusted life years (DALYs) due to silicosis was collected and analyzed. The burden and trends of mortality and DALYs due to silicosis was assessed by 204 countries and territories, by 5-year interval of age group and by sex from 1990 to 2019. And all the regions were divided into 5 categories according to Sociodemographic Index (SDI). Temporal trends in mortality and DALY were evaluated only to ASR by the Joinpoint regression model.

**Results:**

More than 12.9 thousand [95% Uncertainty Intervals (UI): 10.9, 16.2] death cases occurred due to silicosis worldwide, and 655.7 thousand (95% UI: 519.3, 828.0) DALYs were attributed to silicosis in 2019. From 1990 to 2019, global number of mortality and DALYs in countries with high SDI quintile decreased by 0.35% (95% UI: − 0.45, − 0.17) and 0.32% (95% UI: − 0.45, − 0.01), respectively. There was a greater burden in low- and middle-income countries were estimated in 2019 according to ASRs. The global number of mortality and DALYs among males accounted for over 95% of all in 2019. Both age-sex-specific mortality and DALY rate were increasing with aging and reached their peak at 85–89 age group. During the past 30 years, ASR of mortality and DALYs showed a decreasing trend with average annual percentage change at -3.0% [95% Confidence Intervals (CI): − 3.2, − 2.9] and − 2.0 (95% CI: − 1.7, − 2.2), respectively.

**Conclusions:**

Silicosis remains an important health issue and causes a potentially serious burden worldwide. Attention should be paid to making preventable, affordable and effective measures in lower SDI regions.

**Supplementary Information:**

The online version contains supplementary material available at 10.1186/s12890-022-02040-9.

## Background

Silicosis is a major type of pneumoconiosis, caused by inhalation of silica particles or free crystalline silicon dioxide [1], which exist in the air of both living and working environments [2]. Generally, silicosis leads to progressive and almost fatal conditions from the beginning of inflammation and fibrosis [3, 4]. With the irreversible progression of silicosis, severe lung function damage was deteriorating. Various respiratory symptoms are presented including cough, sputum expectoration, shortness of breath, chest tightness, and then worse complications are shown such as tuberculosis, respiratory tract infection, respiratory insufficiency, pneumothorax [1, 5, 6]. Although efforts have been made for several decades, there is no effective treatment for silicosis, except for lung transplantation offered for a minority of patients [7, 8].

Pneumoconiosis remains a public health issue around the world that cause huge health and economic damage [9] in both low-middle income countries such as Russia, China, India [10] and high-income countries [11]. The latest data from the global burden of disease (GBD) study [12], the number of pneumoconiosis cases increased by 66.0% from 1990 to 2017, reaching 60 thousand. And 39% of the cases were ascribed to silicosis globally in 2017, remaining the most crucial risk factor for pneumoconiosis. However, data on expanding the specific knowledge regarding silicosis, updating estimates, and decomposing its burden by age and sex has not been assessed [13–16]. In this study, based on the GBD study 2019, we aimed to comprehensively assess the trends of disease burden due to silicosis worldwide, by gender and age.

## Methods

### Data source

GBD study 2019, covering 369 diseases and injuries in 204 countries and territories from 1990 to 2019 [17], provided comparable and systematic estimations of data by the Institute of Health Metrics and Evaluation. The estimates of pneumoconiosis were predominantly derived from systematic reviews, inpatient hospital reports and reported claims-based data for United States and Taiwan, and then analyzed with demographic methods to complete missing values. A Bayesian meta-regression model (DisMod-MR2.1), as a type of mixed effect model conducted by GBD, controls and adjusts biases of data with information across locations, time and age, to synthesis various sources of data into consistent estimates of levels and trends between parameters [18]. We collected both crude and age-standardized rates (ASR) on mortality and disability-adjusted life years (DALYs) for silicosis, based on online available public data from the Global Health Data Exchange website (http://ghdx.healthdata.org/gbd-results-tool), according to GBD’s operation instruction.

### Definitions

Silicosis belongs to the quaternary level of GBD cause hierarchy, which was defined as a chronic lung disease typified by lung scarring and other interstitial damage caused by exposure to silica [13]. According to Revision of the International Classification of Diseases (ICD) codes, the ICD-10 codes are J62-J62.9 and ICD-9 codes are 502–502.9, and there is no more detailed lower-tier grading in GBD 2019.

The 204 countries and territories were divided into 5 categories according to Sociodemographic Index (SDI): low, low-middle, middle, high-middle, high SDI regions. SDI ranges from 0 to 1, calculated based on the lag-distribution income per capita, education, and total fertility rates [19].

Age-standardized rates (ASR) were generated based on the World Health Organization World Standard Population Distribution, which allowed comparisons across groups with different age demographics compositions. The GBD used both crude and age-standardized rates to describe the burden of silicosis across regions, years and genders.

The GBD computed final estimates by using the mean estimate across 1000 draws, and 95% uncertainty intervals (UI) were determined on the basis of the 25^th^ and 75^th^ percentile values in the draws. It could address the possible heterogeneity from both sampling error and non-sampling variance.

### Statistical analyses

Firstly, we present crude numbers and rates of burden by sex and SDI regions in 1990 and 2019. The global distribution of ASR of burden was depicted by the heat map (R package: ggplot2). Secondly, we applied the population pyramid (R package: plotrix) to show the difference of mortality and DALYs between age and sex in 2019. Lastly, the joinpoint regression model was conducted to evaluate the temporal trend in the ASRs of mortality and DALYs. Based on the Joinpoint regression software version 4.9.0.0 (Version 4.9.0.0 Statistical Research and Application Branch, the United States National Cancer Institute; surveillance.cancer.gov/joinpoint), the best-fitting points were identified by the slope of changing trend and connected a set of statistically linear models on a logarithmic scale [20]. And we determined the number of join points with maximum of 5. The equations of joinpoint regression were listed as follows:

$$E\left[ {y|x} \right] = {\text{e}}^{{\beta_{0} + \beta_{1} x + \delta_{1} \left( {x - \tau_{1} } \right)^{ + } + \cdots + \delta_{k} \left( {x - \tau_{k} } \right)^{ + } }}$$

In this equation, k denotes the number of turning points, $\tau_{k}$ denotes the unknown turning points, $\beta_{0}$ denotes the invariant parameter, $\beta_{1}$ denotes the regression coefficient, $\delta_{k}$ denotes the regression coefficient of the piecewise function of k.

Annual percentage change (APC) and average annual percentage change (AAPC), as well as corresponding 95% CIs, were obtained to quantify the piecewise and overall trends, respectively. We assess whether the APC was significantly different from 0 or not by Z test (Significance hypothesis tests are two-sided and the significance level was ≤ 0.05.). And the remaining analysis and visualization were produced by R studio (version 4.0.3) based on the dataset generated by GDB 2019 study. All methods were carried out in accordance with relevant guidelines and regulations or declaration of Helsinki.

## Results

### Global and national burden and changes

In 2019, there are over 12.9 thousand (95% UI: 10.9, 16.2) deaths due to silicosis worldwide, equivalent to a 0.15% (95% UI: − 0.34, 0.27) decrease compared with the number of deaths in 1990 [15.1 thousand (95% UI: 10.9, 18.5)]. And the corresponding crude rate of mortality decreased by 0.41% (95% UI: − 0.54, − 0.12) from 1990 to 2019 (Table 1). Globally, 655.7 thousand (95% UI: 519.3, 828.0) DALYs were attributed to silicosis in 2019. Comparing to the number of DALYs in 1990 [577.4 thousand (95% UI: 445.9–687.2)], a decrease of 0.15% (95% UI: − 0.34, 0.27) was observed in 2019 (Table 2).

**Table 1:** The number of deaths and crude mortality rates due to silicosis in 1990 and 2019, and changes from 1990 to 2019

Item	1990		2019		Change 1990–2019	
	Number	Rate, per 100,000	Number	Rate, per 100,000	Number	Rate, per 100,000
Global	15,106.77 (10,913.17, 18,493.90)	0.28 (0.20, 0.35)	12,886.69 (10,826.98, 16,160.92)	0.17 (0.14, 0.21)	− 0.15 (− 0.34, 0.27)	0.41 (− 0.54, − 0.12)
*Sex*						
Male	14,697.12 (10,522.5, 18,091.25)	0.55 (0.39, 0.67)	12,335.37 (10,240.87, 15,401.57)	0.32 (0.26, 0.40)	0.12 (− 0.10, 0.46)	− 0.22 (− 0.38, 0.01)
Female	409.64 (293.29, 698.02)	0.02 (0.01, 0.03)	551.31 (408.01, 724.08)	0.01 (0.01, 0.02)	0.49 (0.07, 0.78)	0.03 (− 0.26, 0.22)
SDI*						
Low	364.24 (57.57, 706.08)	0.07 (0.01, 0.13)	475.29 (127.92, 783.54)	0.04 (0.01, 0.07)	0.32 (− 0.05, 1.17)	− 0.38 (− 0.55, 0.01)
Low-middle	1996.52 (862.5, 2870.52)	0.18 (0.08, 0.25)	2195.08 (1461.46, 2866.11)	0.12 (0.08, 0.16)	0.23 (− 0.05, 0.83)	− 0.21 (− 0.39, 0.17)
Middle	5537.77 (3615.10, 7454.95)	0.32 (0.21, 0.43)	5482.36 (4242.18, 7131.65)	0.23 (0.18, 0.30)	0.28 (− 0.03, 0.76)	− 0.08 (− 0.30, 0.26)
High-middle	4764.88 (3835.70, 5525.29)	0.41 (0.33, 0.48)	3084.46 (2568.01, 4639.50)	0.22 (0.18, 0.32)	0.04 (− 0.16, 0.28)	− 0.17 (− 0.32, 0.03)
High	2440.56 (2234.99, 2758.73)	0.30 (0.27, 0.34)	1647.64 (1328.14, 2829.05)	0.16 (0.13, 0.28)	− 0.35 (− 0.45, − 0.17)	− 0.47 (− 0.55, − 0.33)

The table were presented with values (95% uncertainty intervals)

*SDI: Sociodemographic index

**Table 2:** The number of DALYs and crude DALY rates due to silicosis in 1990 and 2019, and changes from 1990 to 2019

Item	1990		2019		Change 1990–2019	
	Number	Rate, per 100,000	Number	Rate, per 100,000	Number	Rate, per 100,000
Global	577,390.01 (445,865.09, 709,168.89)	10.79 (8.33, 13.26)	655,762.89 (519,296.99, 828,025.13)	8.48 (6.71, 10.70)	− 0.15 (− 0.34, 0.27)	− 0.41 (− 0.54, − 0.12)
*Sex*						
Male	559,290.43 (427,616.53, 687,236.82)	0.04 (0.03, 0.05)	628,707.83 (496,793.24, 800,453.04)	16.20 (12.80, 20.63)	− 0.16 (− 0.35, 0.27)	− 0.42 (− 0.55, − 0.12)
Female	18,099.58 (13,394.70, 25,965.38)	0.68 (0.50, 0.98)	27,055.06 (20,663.40, 36,038.87)	0.70 (0.54, 0.93)	0.35 (− 0.23, 0.83)	− 0.07 (− 0.47, 0.26)
SDI*						
Low	10,079.05 (2396.22, 18,956.52)	1.91 (0.45, 3.59)	13,282.91 (4999.40, 21,030.17)	1.18 (0.44, 1.86)	0.30 (− 0.06, 1.35)	− 0.39 (− 0.56, 0.10)
Low-middle	68,609.10 (38,985.81, 91,393.72)	6.07 (3.45, 8.09)	84,548.38 (62,997.10, 106,296.92)	4.79 (3.57, 6.03)	0.10 (− 0.17, 0.96)	− 0.30 (− 0.47, 0.26)
Middle	255,801.89 (185,145.05, 329,313.36)	14.90 (10.78, 19.18)	328,414.92 (253,904.04, 424,079.48)	13.7 (10.59, 17.7)	− 0.01 (− 0.35, 0.77)	− 0.29 (− 0.53, 0.27)
High-middle	185,017.51 (149,262.20, 223,147.50)	16.08 (12.97, 19.4)	192,031.88 (144,494.98, 251,436.87)	13.43 (10.1, 17.58)	− 0.35 (− 0.50, − 0.01)	− 0.48 (− 0.60, − 0.21)
High	57,818.48 (51,692.25, 66,470.10)	7.03 (6.29, 8.09)	37,443.40 (30,548.20, 53,827.24)	3.69 (3.01, 5.31)	− 0.32 (− 0.45, − 0.01)	− 0.45 (− 0.55, − 0.19)

The table were presented with values (95% uncertainty intervals)

*SDI: Sociodemographic index

Middle-SDI countries had highest numbers [5.5 thousand (95% UI: 4.2, 7.1)] and crude rate [0.23 (95% UI: 0.18, 0.30)] of mortality in 2019. From 1990 to 2019, global deaths increased by 0.32% (95% UI: 0.26, 0.4) in countries with low SDI quintile, while countries with high SDI quintile showed a decreasing number of mortality [− 0.35% (95% UI: − 0.45, − 0.17)] (Table 1). Middle-SDI countries had highest numbers [328.4 thousand (95% UI: 253.9, 424.1)] and crude rate [13.70 (95% UI: 10.59, 17.70)] of DALYs in 2019.The greater reduction in number of DALYs change was seen in countries with high-middle and high SDI quintiles [− 0.35% (95% UI: − 0.5, − 0.01) and − 0.32% (95% UI: − 0.45, − 0.01), respectively] (Table 2).

**Fig. 1:**
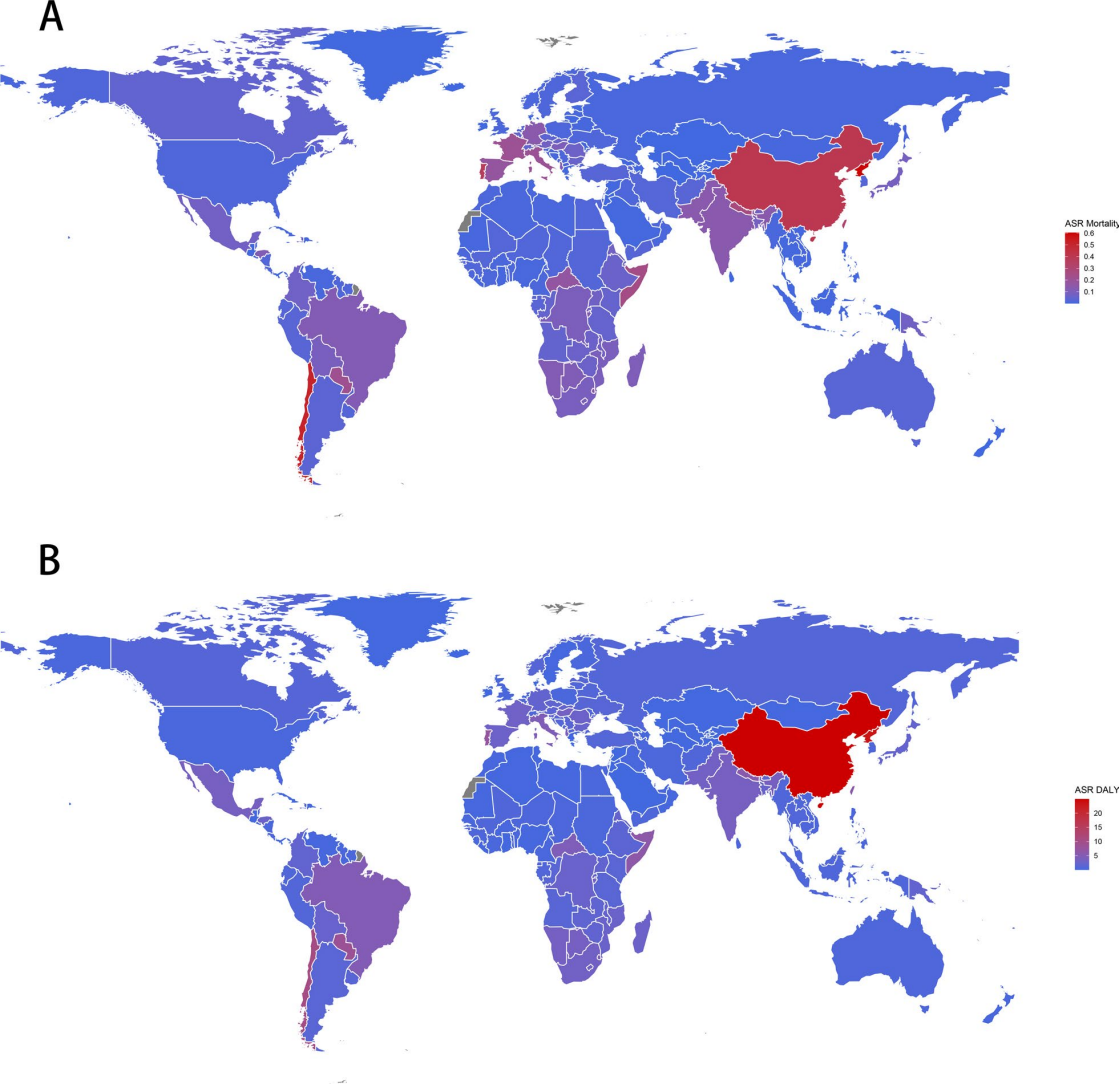
Global age standardized **a** mortality, **b** DALY rate per 100,000 of silicosis for both sexes in 2019

The low- and middle-income countries: Palau, North Korea, Chile, China, and Portugal ranked in the top 5 according to ASRs for mortality in 2019. China has the highest ASRs for DALY due to silicosis in 2019 across the world [24.97 per 100,000 people (95% UI: 18.90, 32.51)], followed by North Korea, Palau, and Chile where rates were estimated to exceed 10 per 100,000 people (Fig. 1). And the detailed ASRs for mortality and DALY due to silicosis in 2019 were presented in Additional file 1: Table S1.

### Age- and sex-specific burden in 2019

In 2019, there were 12.3 thousand (95% UI: 10.2, 15.4) deaths among males, and it took up 95.72% of all the deaths (Table 1). The crude rates of DLAY in males [16.2 per 100,000 people (95% UI: 12.8, 20.63)] were outweighing those in females [0.7 per 100,000 (95% UI: 0.54, 0.93) people] in 2019, reflecting the sex ratio for mortality remained high (Table 2).

**Fig. 2:**
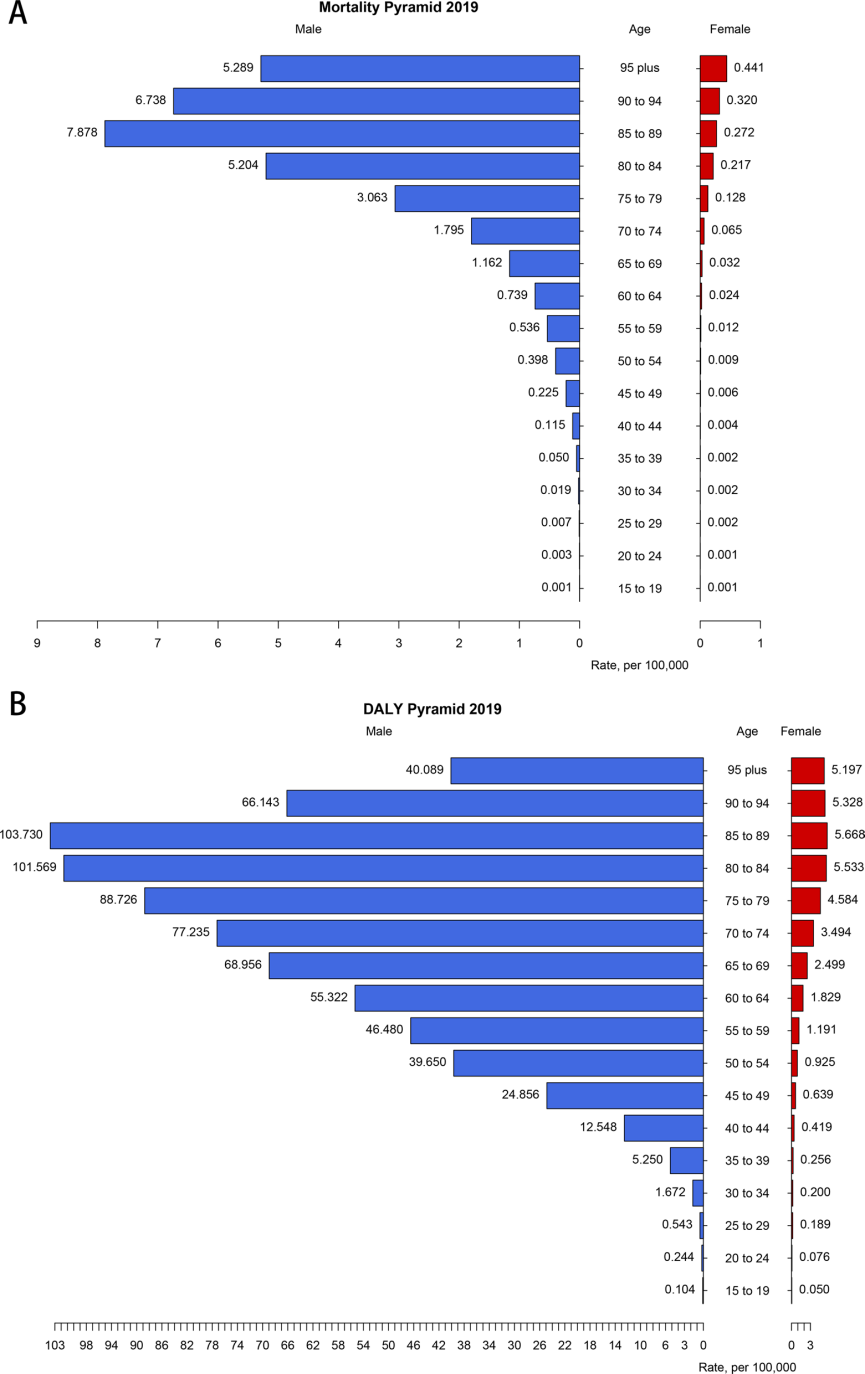
Global **a** mortality, **b** DALY rate per 100,000 of silicosis by age and sex in 2019

The age-sex-specific mortality of silicosis in 2019 increased with aging generally. Both mortality and DALY rates peaked at 85–89 age group among males [7.9 per 100,000 people (95% UI: 6.6, 10.5) and 103.7 per 100,000 people (95% UI: 88.4, 126.7)], and the rates were much greater in males than females across all age groups. From 65 years old onwards in males, the mortality rates of silicosis were estimated over 1 per 100,000 and presented an exponential growth. However, from the onset of 30–90 years old, the DALY rates due to silicosis increased monotonically at a steady pace. For mortality rate of silicosis among females, it didn’t show a substantial upward until 65 years old. DALY rates among females stopped increasing until 90 years old (Fig. 2). Detailed age- and sex-specific burden due to silicosis were presented in Additional file 1: Table S2.

### Global trends of silicosis by sex

**Fig. 3:**
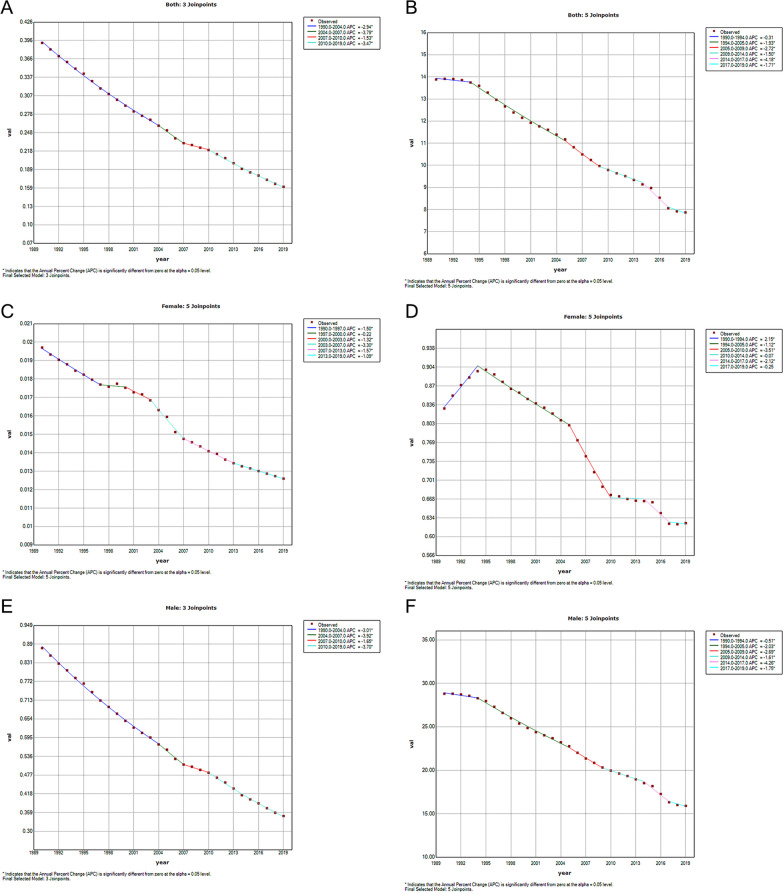
Global Trend of ASR (**a**: both sexes, **c**: female, **e**: male) mortality, (**b**: both sexes, **d**: female, **f**: male) DALY per 100,000 of silicosis by Joinpoint Regression, 1990–2019

**Fig. 4:**
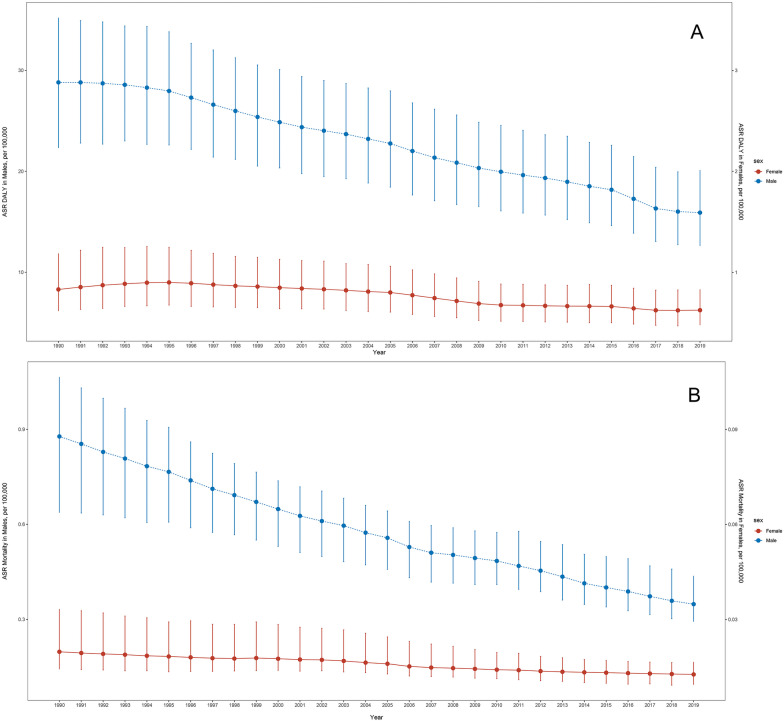
Global Trend of ASR **a** mortality, **b** DALY per 100,000 of silicosis by sex from 1990 to 2019

Joinpoint regression analysis identified trends in ASRs due to silicosis by sex from 1990 to 2019. For overall ASRs for mortality, the decreasing trend across the study period was observed, with AAPC at − 3.0% (95% CI: − 3.2, − 2.9). Apart from males, ASRs for mortality among females had a variable trending during the 30 years. The decreasing trend wasn’t observed significantly (APC =  − 0.2%, 95% CI: − 1.4, 1.0) from 1997 to 2000, although the rest of the period descended with a different slope. As for ASR DALY overall, there is a relatively stable period for the first 4 years of observation (APC =  − 0.3%, 95% CI: − 0.8, 0.2), and it had gone through a drastically descending from 2014 to 2017 (APC =  − 4.7%, 95% CI: − 5.7, − 2.7). From 1990 to 1994, ASR DALY had an increased slope at 1.9% (95% CI: 2.4, 19.5) among females. A continuous declination occurred during the whole study period in ASR DALY among males [AAPC = -2.1%, (95% CI: -1.8, -18.2)], almost twice as many as females (Figs. 3 and 4). Detailed information was recorded in Additional file 1: Tables S3 and S4.

## Discussion

This study reported recent trends of the numbers and ASRs of mortality and DALY due to silicosis by region, age and sex over the last three decades. Using GBD 2019 data, this study found that while mortality and DALYs due to silicosis have shown an overall decreasing trend since 1990, countries with low SDI faced a heavier disease burden. Compared to females, males with silicosis had higher mortality and DALY rates at all ages. Notably, from 1990 to 2019, the disease burden for both males and females with silicosis showed an overall decreasing trend, more pronounced in males.

There are some differences in the disease burden of silicosis between patients in terms of gender. In the past, the traditional perception was that males’ health was affected by their occupation and suffered more damage from silicosis. Statistically, ASR for mortality also showed a decrease in the number of males and a constant trend in females. However, in terms of actual deaths, the number of female mortality and DALYs were on the increase against a background of decreasing annual deaths in males with silicosis. This leads us to take into account the impact of silicosis on females. Firstly, females in traditional occupations have the same potential for silicosis exposure as males [21]. Secondly, various emerging occupations result in males and females having an equal chance of being damaged by silicosis, such as jeans sandblasting, jewelry polishing, and other emerging occupations [10, 22, 23]. Finally, there are also potential gender differences in the diagnosis of silicosis for females due to gender bias [24]. Fortunately, the findings still showed that the main impact of the disease burden of silicosis occurs in older. Both mortality rates and DALYs showed lower levels in the younger age groups. Silicosis did not appear to be a large-scale acute or accelerated disease [25].

In terms of healthcare resource consumption, the consumption of resources for silicosis patients had also decreased in recent years compared to the 1990s [26]. Based on the early recognition of the dangers of silicosis, various measures have been taken all over the world to prevent and treat silicosis. However, it could not be ignored that we still face a very serious silicosis problem. On the one hand, workers in low- and middle-income countries are still at risk from traditional occupational exposure [2]. China and South Africa, for example, are countries where the main source of silicosis is the mining industry. These countries still face a high burden of disease. On the other hand, high-income countries are inevitably affected by silicosis. In Spain, for example [27], occupational exposures such as secondary processing of quartz processing led to the re-emergence of silicosis patients; Since 2000, the popularity of new artificial stone has led to silicosis among workers in European countries [28]. The main reason for these occurrences is that the main risk factors for silicosis have not changed significantly. Occupational exposure has remained the main risk factor for silicosis for the past decade. Exposure is very common in low- and middle-income countries [2]. The lack of early detection of silicosis leaves us with a continuing need for exploration when confronted with silicosis [29]. The results suggest that although some preventive measures for silicosis are known, exposure to risk factors has been ignored, leading to the development of silicosis [30] and an increase in the number of deaths.

The reason for this problem is that silicosis can lead to serious consequences. For people with silicosis, the presence of silicosis can lead to the development of diseases such as lung cancer [31]. In addition, even after exposure has ceased, silicosis can gradually affect the individual's health. For medical researchers, the prevention and treatment of silicosis were still based on simulations of animal models and limited exploration of the affected population [32]. Silicosis remains an important occupational health problem, especially in low- and middle-income countries [33]. Despite the many difficulties in the fight against silicosis, some efforts were moving in a better direction. From a group perspective, there has been a gradual increase in concern about silicosis. Media coverage and internet search behavior for silicosis have increased significantly [34]. At the national level, the prevention of silicosis has also been explored, for example by adding silicosis to the nationwide surveillance as part of the National Public Health Surveillance System [35]. It is recommended that countries pay attention to the prevention of silicosis and invest more in research. Occupational exposure limits should be set out in legal provisions and mandatory restrictions should be implemented in all sectors where silicosis exposure occurs.

There are some limitations to this study. Firstly, similar to most GBD studies, the robustness of the results is affected by the potential quality of the raw data. Secondly, this study was unable to estimate the situation of people with silicosis that was masked by other lung diseases, and the true burden of silicosis may be underestimated. Finally, these data were based on calculations of surveillance data, and these indicators may have a lag in the estimation of the actual disease burden.

## Conclusion

Silicosis continues to be one of the most important occupational health issue and causes a potentially serious disease burden worldwide. Although the ASR of mortality and DALY for silicosis have trended downwards globally from 1990 to 2019, they have increased in women and in low- and middle-income countries. Therefore, attention should be paid to the disease burden in lower SDI regions, and preventable, affordable and effective measures are urgent to make globally.

## Supplementary Information

**Additional file 1.** **Table S1.** Global age-standardized mortality and DALY rate of silicosis for both sexes in 2019. 204 countries are arranged by GBD 7 super regions and 21 regions ASR: age-standardized rate; DALY: disability-adjusted life years. **Table S2.** Global age- and sex-specific rate for mortality and DALY due to silicosis in 2019. DALY: disability-adjusted life years. **Table S3.** Global trend of age-standardized mortality and DALY rates of silicosis by Joinpoint regression, 1990-2019. APC: annual percentage change; CI: confidential interval; AAPC: average annual percentage change.

## Data Availability

The datasets are publicly available in the IHME Data (http://ghdx.healthdata.org/gbd-results-tool).

## Abbreviations

GBD: Global Burden of Disease
DALY: Disability-adjusted life year
ICD: International classification of diseases
SDI: Sociodemographic index
ASR: Age-standardized rates
UI: Uncertainty intervals
APC: Annual percentage change
AAPC: Average annual percentage change
