# Supplementary material for: Global and national burden and trends of mortality and disability-adjusted life years for silicosis, from 1990 to 2019: results from the Global Burden of Disease study 2019
Source: BMC Pulm Med. 2022 Jun 21;22:240. doi: 10.1186/s12890-022-02040-9 (PMC9210623; doi:10.1186/s12890-022-02040-9)
Supplement: Supplementary file 1 — Additional file 1. Table S1. Global age-standardized mortality and DALY rate of silicosis for both sexes in 2019. 204 countries are arranged by GBD 7 super regions and 21 regions ASR: age-standardized rate; DALY: disability-adjusted life years. Table S2. Global age- and sex-specific rate for mortality and DALY due to silicosis in 2019. DALY: disability-adjusted life years. Table S3. Global trend of age-standardized mortality and DALY rates of silicosis by Joinpoint regression, 1990-2019. APC: annual percentage change; CI: confidential interval; AAPC: average annual percentage change. [file 12890_2022_2040_MOESM1_ESM.docx]

# Supplementary File

## Supplementary table 1 Global age-standardized mortality and DALY rate of silicosis for both sexes in 2019

| Location | ASR Mortality, per 100,000 | ASR DALY, per 100,000 |
| --- | --- | --- |
| Central Europe, Eastern Europe, and Central Asia | 0.0199 (0.0169, 0.0260) | 1.0634 (0.8059, 1.4388) |
| Central Asia | 0.0047 (0.0036, 0.0069) | 0.2126 (0.1530, 0.2938) |
| Armenia | 0.0257 (0.0192, 0.0343) | 0.6636 (0.5048, 0.8973) |
| Azerbaijan | 0.0055 (0.0028, 0.0113) | 0.2570 (0.1703, 0.3791) |
| Georgia | 0.0024 (0.0013, 0.0040) | 0.1568 (0.1002, 0.2347) |
| Kazakhstan | 0.0009 (0.0004, 0.0029) | 0.1389 (0.0857, 0.2137) |
| Kyrgyzstan | 0.0003 (0.0002, 0.0004) | 0.1355 (0.0795, 0.2121) |
| Mongolia | 0.0074 (0.0019, 0.0243) | 0.2386 (0.1329, 0.5423) |
| Tajikistan | 0.0014 (0.0008, 0.0024) | 0.1734 (0.1095, 0.2592) |
| Turkmenistan | 0.0007 (0.0004, 0.0015) | 0.1474 (0.0906, 0.2270) |
| Uzbekistan | 0.0036 (0.0023, 0.0057) | 0.1980 (0.1347, 0.2866) |
| Central Europe | 0.0392 (0.0326, 0.0500) | 1.5763 (1.2517, 2.0080) |
| Albania | 0.1153 (0.0647, 0.2177) | 3.9944 (2.5915, 6.0976) |
| Bosnia and Herzegovina | 0.0071 (0.0031, 0.0130) | 0.9054 (0.5531, 1.4165) |
| Bulgaria | 0.0541 (0.0355, 0.0872) | 2.1315 (1.5487, 2.9300) |
| Croatia | 0.0072 (0.0034, 0.0297) | 1.1712 (0.7386, 1.7797) |
| Czechia | 0.0374 (0.0247, 0.0534) | 1.6611 (1.1729, 2.3010) |
| Hungary | 0.1021 (0.0774, 0.1282) | 3.2335 (2.5082, 4.1188) |
| Montenegro | 0.021 (0.01140, 0.0363) | 1.1854 (0.7882, 1.6964) |
| North Macedonia | 0.0121 (0.0086, 0.0171) | 1.0796 (0.7360, 1.5737) |
| Poland | 0.0144 (0.0109, 0.0201) | 0.5051 (0.3987, 0.6373) |
| Romania | 0.0650 (0.0492, 0.0840) | 2.5307 (1.9018, 3.2712) |
| Serbia | 0.0149 (0.0098, 0.0261) | 1.1532 (0.7776, 1.7003) |
| Slovakia | 0.0551 (0.0393, 0.0749) | 2.1048 (1.5325, 2.8239) |
| Slovenia | 0.0086 (0.0035, 0.0519) | 1.5820 (0.9138, 2.5575) |
| Eastern Europe | 0.0107 (0.0085, 0.0159) | 0.9708 (0.6707, 1.4115) |
| Belarus | 0.0077 (0.0043, 0.0154) | 0.9124 (0.5839, 1.4173) |
| Estonia | 0.002 (0.00140, 0.0029) | 0.8339 (0.4880, 1.3281) |
| Latvia | 0.0097 (0.0071, 0.0132) | 1.0733 (0.6969, 1.6195) |
| Lithuania | 0.0027 (0.0018, 0.0039) | 0.7966 (0.5151, 1.2004) |
| Republic of Moldova | 0.0012 (0.0009, 0.0017) | 0.8164 (0.4741, 1.2907) |
| Russian Federation | 0.0094 (0.0074, 0.0153) | 0.9154 (0.6184, 1.3307) |
| Ukraine | 0.0171 (0.0115, 0.0247) | 1.1787 (0.8097, 1.7046) |
| High-income | 0.0879 (0.0736, 0.1294) | 1.6894 (1.4445, 2.2636) |
| Australasia | 0.0251 (0.0145, 0.0814) | 0.5990 (0.4081, 1.4294) |
| Australia | 0.0295 (0.0170, 0.0958) | 0.6456 (0.4287, 1.6164) |
| New Zealand | 0.0013 (0.0007, 0.0028) | 0.3508 (0.2087, 0.5389) |
| High-income Asia Pacific | 0.0756 (0.0531, 0.1499) | 1.8073 (1.3715, 2.8844) |
| Brunei Darussalam | 0.0085 (0.0055, 0.0145) | 0.3941 (0.2679, 0.5666) |
| Japan | 0.0850 (0.0607, 0.1585) | 2.0319 (1.5660, 2.9725) |
| Republic of Korea | 0.0252 (0.0100, 0.1222) | 0.7789 (0.4288, 2.5214) |
| Singapore | 0.0048 (0.0032, 0.0062) | 0.3980 (0.2559, 0.5847) |
| High-income North America | 0.0201 (0.0168, 0.0338) | 0.5338 (0.4509, 0.7398) |
| Canada | 0.0439 (0.0289, 0.1066) | 1.0314 (0.7823, 1.9148) |
| Greenland | 0.0020 (0.0012, 0.0046) | 0.1436 (0.0962, 0.2143) |
| United States of America | 0.0172 (0.0146, 0.0241) | 0.4734 (0.3981, 0.5795) |
| Georgia | 0.0024 (0.0013, 0.0040) | 0.1568 (0.1002, 0.2347) |
| Southern Latin America | 0.1658 (0.1404, 0.2016) | 3.8794 (3.3248, 4.5160) |
| Argentina | 0.0338 (0.0268, 0.0411) | 1.3034 (1.0323, 1.6245) |
| Chile | 0.5107 (0.4196, 0.6346) | 10.4241 (8.7344, 12.5513) |
| Uruguay | 0.0238 (0.0194, 0.0284) | 1.2367 (0.9632, 1.5553) |
| Western Europe | 0.1309 (0.1124, 0.1723) | 2.1921 (1.8990, 2.7952) |
| Andorra | 0.0382 (0.0103, 0.1412) | 0.5704 (0.1635, 2.1332) |
| Austria | 0.0771 (0.0617, 0.1012) | 1.3827 (1.1107, 1.7647) |
| Belgium | 0.0507 (0.0361, 0.0697) | 0.8242 (0.5861, 1.1362) |
| Cyprus | 0.0259 (0.0172, 0.0450) | 0.3766 (0.2688, 0.5968) |
| Denmark | 0.0046 (0.0032, 0.0081) | 0.0833 (0.0579, 0.1510) |
| Finland | 0.0313 (0.0161, 0.1332) | 0.5042 (0.2747, 1.9932) |
| France | 0.2077 (0.1535, 0.2931) | 2.8800 (2.1784, 4.1083) |
| Germany | 0.1395 (0.1060, 0.2047) | 1.8860 (1.4527, 2.9390) |
| Greece | 0.0046 (0.0035, 0.0059) | 0.0944 (0.0695, 0.1302) |
| Iceland | 0.0012 (0.0006, 0.0017) | 0.0234 (0.0126, 0.0321) |
| Ireland | 0.0037 (0.0020, 0.0115) | 0.0849 (0.0459, 0.2415) |
| Israel | 0.0158 (0.0127, 0.0201) | 0.4734 (0.3772, 0.5971) |
| Italy | 0.2026 (0.1735, 0.2286) | 4.4688 (3.7893, 5.3630) |
| Luxembourg | 0.0692 (0.0513, 0.0919) | 1.2206 (0.9057, 1.6205) |
| Malta | 0.0023 (0.0014, 0.0050) | 0.0396 (0.0254, 0.0821) |
| Monaco | 0.0917 (0.0516, 0.1589) | 1.5662 (0.8813, 2.7299) |
| Netherlands | 0.0287 (0.0206, 0.0490) | 0.4318 (0.3162, 0.7344) |
| Norway | 0.0120 (0.0096, 0.0218) | 1.0944 (0.7538, 1.5609) |
| Portugal | 0.3662 (0.3047, 0.4386) | 7.2286 (5.9976, 8.7698) |
| San Marino | 0.0845 (0.0501, 0.1323) | 1.3669 (0.8068, 2.1346) |
| Spain | 0.1766 (0.1384, 0.2344) | 2.6483 (2.1396, 3.4727) |
| Sweden | 0.0151 (0.0101, 0.0337) | 0.2519 (0.1865, 0.4616) |
| Switzerland | 0.0456 (0.0348, 0.0625) | 0.6356 (0.4954, 0.8451) |
| United Kingdom | 0.0123 (0.0094, 0.0246) | 0.4689 (0.3525, 0.6923) |
| Latin America and Caribbean | 0.0774 (0.0701, 0.0863) | 3.0773 (2.6171, 3.6466) |
| Andean Latin America | 0.0368 (0.0236, 0.0593) | 1.0553 (0.7115, 1.5994) |
| Bolivia (Plurinational State of) | 0.0912 (0.0467, 0.1443) | 2.2514 (1.2770, 3.4749) |
| Ecuador | 0.0248 (0.0158, 0.0398) | 0.7567 (0.5176, 1.1718) |
| Peru | 0.0289 (0.0143, 0.0592) | 0.8697 (0.5140, 1.5504) |
| Caribbean | 0.0131 (0.0074, 0.0235) | 0.3132 (0.1711, 0.5634) |
| Antigua and Barbuda | 0.0018 (0.0011, 0.0025) | 0.0456 (0.0297, 0.0645) |
| Bahamas | 0.0345 (0.0256, 0.0460) | 0.8342 (0.6269, 1.0996) |
| Barbados | 0.0028 (0.0020, 0.0038) | 0.0597 (0.0451, 0.0767) |
| Belize | 0.0104 (0.0075, 0.0143) | 0.3436 (0.2266, 0.5121) |
| Bermuda | 0.0405 (0.0310, 0.0511) | 0.7892 (0.6155, 0.9998) |
| Cuba | 0.0097 (0.0071, 0.0126) | 0.1807 (0.1333, 0.2406) |
| Dominica | 0.0054 (0.0031, 0.0083) | 0.1271 (0.0732, 0.1899) |
| Dominican Republic | 0.0051 (0.0030, 0.0088) | 0.1269 (0.0763, 0.2219) |
| Grenada | 0.0074 (0.0053, 0.0100) | 0.1533 (0.1140, 0.2040) |
| Guyana | 0.0184 (0.0132, 0.0251) | 0.4717 (0.3403, 0.6402) |
| Haiti | 0.0580 (0.0102, 0.1461) | 1.1656 (0.2303, 2.8762) |
| Jamaica | 0.0033 (0.0021, 0.0048) | 0.0698 (0.0471, 0.1013) |
| Puerto Rico | 0.0013 (0.0008, 0.0019) | 0.0323 (0.0227, 0.0473) |
| Saint Kitts and Nevis | 0.0067 (0.0048, 0.0089) | 0.1582 (0.1105, 0.2168) |
| Saint Lucia | 0.0075 (0.0052, 0.0102) | 0.1754 (0.1273, 0.2337) |
| Saint Vincent and the Grenadines | 0.02 (0.014200, 0.0267) | 0.4841 (0.3520, 0.6371) |
| Suriname | 0.0199 (0.0114, 0.0311) | 0.5190 (0.2925, 0.8092) |
| Trinidad and Tobago | 0.0023 (0.0015, 0.0035) | 0.0598 (0.0395, 0.1021) |
| United States Virgin Islands | 0.0070 (0.0042, 0.0109) | 0.1558 (0.0928, 0.2494) |
| Central Latin America | 0.058 (0.04790, 0.0692) | 2.2939 (1.8340, 2.8283) |
| Colombia | 0.0695 (0.0497, 0.0939) | 1.8486 (1.3584, 2.4436) |
| Costa Rica | 0.0181 (0.0126, 0.0253) | 0.6138 (0.4464, 0.8373) |
| El Salvador | 0.0052 (0.0034, 0.0075) | 0.2309 (0.1630, 0.3111) |
| Guatemala | 0.0036 (0.0014, 0.0049) | 0.1823 (0.1208, 0.2525) |
| Honduras | 0.1132 (0.0350, 0.1959) | 2.5501 (0.8795, 4.3651) |
| Mexico | 0.0755 (0.0602, 0.0937) | 3.5015 (2.7322, 4.4461) |
| Nicaragua | 0.0218 (0.0161, 0.0299) | 0.4943 (0.3705, 0.6529) |
| Panama | 0.0030 (0.0020, 0.0051) | 0.1727 (0.1202, 0.2460) |
| Venezuela (Bolivarian Republic of) | 0.0095 (0.0064, 0.0133) | 0.3241 (0.2363, 0.4397) |
| Tropical Latin America | 0.1198 (0.1092, 0.1349) | 4.9129 (4.1735, 5.8721) |
| Brazil | 0.1173 (0.1067, 0.1334) | 4.8203 (4.0726, 5.8065) |
| Paraguay | 0.2195 (0.1406, 0.3083) | 8.4995 (5.6658, 11.602) |
| North Africa and Middle East | 0.0185 (0.0101, 0.0264) | 0.4940 (0.2900, 0.6929) |
| North Africa and Middle East | 0.0185 (0.0101, 0.0264) | 0.4940 (0.2900, 0.6929) |
| Afghanistan | 0.0295 (0.0062, 0.0580) | 0.7493 (0.1770, 1.4555) |
| Algeria | 0.0109 (0.0038, 0.0183) | 0.2433 (0.1086, 0.3850) |
| Bahrain | 0.0050 (0.0024, 0.0177) | 0.1583 (0.0937, 0.3608) |
| Egypt | 0.0200 (0.0042, 0.0401) | 0.5179 (0.1380, 1.0202) |
| Iran (Islamic Republic of) | 0.0233 (0.0093, 0.0304) | 0.5373 (0.2580, 0.6723) |
| Iraq | 0.0160 (0.0088, 0.0269) | 0.3791 (0.2042, 0.6168) |
| Jordan | 0.0018 (0.0011, 0.0026) | 0.0616 (0.0438, 0.0852) |
| Kuwait | 0.0027 (0.0016, 0.0039) | 0.0797 (0.0561, 0.1086) |
| Lebanon | 0.0102 (0.0031, 0.0274) | 0.2597 (0.0989, 0.6338) |
| Libya | 0.0103 (0.0035, 0.0180) | 0.2627 (0.1009, 0.4451) |
| Morocco | 0.0140 (0.0044, 0.0237) | 0.3283 (0.1171, 0.5516) |
| Oman | 0.0121 (0.0037, 0.0229) | 0.2616 (0.0976, 0.4611) |
| Palestine | 0.0031 (0.0021, 0.0047) | 0.0811 (0.0586, 0.1101) |
| Qatar | 0.0057 (0.0033, 0.0095) | 0.1208 (0.0812, 0.1825) |
| Saudi Arabia | 0.0045 (0.0023, 0.0075) | 0.1214 (0.0764, 0.1801) |
| Sudan | 0.0218 (0.0043, 0.0452) | 0.5324 (0.1297, 1.0922) |
| Syrian Arab Republic | 0.0043 (0.0020, 0.0070) | 0.1084 (0.0623, 0.1617) |
| Tunisia | 0.0095 (0.0031, 0.0171) | 0.2293 (0.0951, 0.3978) |
| Turkey | 0.0270 (0.0174, 0.0521) | 0.9760 (0.6521, 1.5816) |
| United Arab Emirates | 0.0164 (0.0025, 0.0587) | 0.4671 (0.1081, 1.4705) |
| Yemen | 0.0257 (0.0066, 0.0487) | 0.6377 (0.1832, 1.1794) |
| South Asia | 0.1343 (0.0598, 0.1983) | 3.4332 (1.7836, 4.8802) |
| South Asia | 0.1343 (0.0598, 0.1983) | 3.4332 (1.7836, 4.8802) |
| Bangladesh | 0.0844 (0.0187, 0.1499) | 2.2059 (0.8907, 3.6221) |
| Bhutan | 0.0825 (0.0172, 0.1566) | 1.9061 (0.6584, 3.2768) |
| India | 0.1373 (0.0611, 0.2087) | 3.5597 (1.9081, 5.1010) |
| Nepal | 0.2691 (0.0593, 0.4780) | 5.6840 (1.5376, 10.1216) |
| Pakistan | 0.1377 (0.0526, 0.2136) | 3.1457 (1.3488, 4.8379) |
| Southeast Asia, East Asia, and Oceania | 0.3166 (0.2408, 0.4344) | 18.9654 (14.3893, 24.5219) |
| East Asia | 0.4058 (0.3171, 0.5561) | 24.6103 (18.6496, 31.8515) |
| China | 0.4039 (0.3122, 0.5635) | 24.9694 (18.8997, 32.5135) |
| Democratic People's Republic of Korea | 0.5987 (0.2711, 1.0491) | 23.475 (13.4576, 36.796) |
| Taiwan (Province of China) | 0.3078 (0.2132, 0.4151) | 5.8963 (4.2461, 7.7258) |
| Oceania | 0.0590 (0.0167, 0.1250) | 1.6985 (0.9232, 2.8309) |
| American Samoa | 0.0034 (0.0021, 0.0064) | 0.7665 (0.4448, 1.1670) |
| Cook Islands | 0.0079 (0.0042, 0.0153) | 0.8246 (0.5102, 1.2306) |
| Fiji | 0.0021 (0.0013, 0.0036) | 0.7241 (0.4262, 1.1189) |
| Guam | 0.0016 (0.0009, 0.0042) | 0.7546 (0.4519, 1.1826) |
| Kiribati | 0.2114 (0.1113, 0.3497) | 4.2668 (2.6869, 6.4454) |
| Marshall Islands | 0.0425 (0.0149, 0.0900) | 1.3919 (0.8384, 2.1252) |
| Micronesia (Federated States of) | 0.0393 (0.0167, 0.0784) | 1.3438 (0.8213, 2.0832) |
| Nauru | 0.0321 (0.0164, 0.0556) | 1.1758 (0.7906, 1.6513) |
| Niue | 0.0157 (0.0084, 0.0274) | 0.9085 (0.5941, 1.3185) |
| Northern Mariana Islands | 0.0034 (0.0016, 0.0106) | 0.8013 (0.4854, 1.2541) |
| Palau | 0.6041 (0.3600, 1.0134) | 13.1142 (7.9614, 20.6269) |
| Papua New Guinea | 0.0738 (0.0143, 0.1677) | 1.8981 (0.9011, 3.4192) |
| Samoa | 0.0307 (0.0169, 0.0571) | 1.1748 (0.7838, 1.6814) |
| Solomon Islands | 0.0860 (0.0224, 0.1784) | 2.1638 (1.0898, 3.7071) |
| Tokelau | 0.0183 (0.0090, 0.0333) | 0.9964 (0.6521, 1.4523) |
| Tonga | 0.0238 (0.0111, 0.0472) | 1.0221 (0.6474, 1.5377) |
| Tuvalu | 0.0319 (0.0130, 0.0654) | 1.1651 (0.7289, 1.7571) |
| Vanuatu | 0.0580 (0.0134, 0.1235) | 1.6785 (0.8741, 2.8096) |
| Southeast Asia | 0.0049 (0.0025, 0.0085) | 0.7057 (0.4692, 1.0124) |
| Cambodia | 0.0061 (0.0024, 0.0119) | 0.6602 (0.4248, 0.9791) |
| Indonesia | 0.0052 (0.002, 0.01200) | 0.7090 (0.4695, 1.0490) |
| Lao People's Democratic Republic | 0.0078 (0.0033, 0.0185) | 0.7792 (0.4879, 1.2000) |
| Malaysia | 0.0032 (0.0020, 0.0053) | 0.7412 (0.4555, 1.1645) |
| Maldives | 0.0052 (0.0027, 0.0079) | 0.9241 (0.5922, 1.3741) |
| Mauritius | 0.0012 (0.0008, 0.0017) | 0.6387 (0.3638, 1.0378) |
| Myanmar | 0.0108 (0.0037, 0.0196) | 0.7702 (0.4858, 1.1386) |
| Philippines | 0.0038 (0.0023, 0.0055) | 0.6067 (0.3981, 0.8834) |
| Seychelles | 0.0028 (0.0009, 0.0093) | 0.6798 (0.4080, 1.0705) |
| Sri Lanka | 0.0011 (0.0006, 0.0019) | 0.6216 (0.3594, 0.9681) |
| Thailand | 0.0046 (0.0028, 0.0073) | 0.8029 (0.5201, 1.1992) |
| Timor-Leste | 0.0070 (0.0026, 0.0153) | 0.7823 (0.4942, 1.1881) |
| Viet Nam | 0.0053 (0.0019, 0.0103) | 0.6703 (0.4176, 1.0211) |
| Sub-Saharan Africa | 0.0522 (0.0211, 0.0873) | 1.4299 (0.6997, 2.2772) |
| Central Sub-Saharan Africa | 0.0862 (0.0175, 0.1929) | 2.1182 (0.5444, 4.5675) |
| Angola | 0.0469 (0.0146, 0.0982) | 1.2252 (0.4956, 2.4064) |
| Central African Republic | 0.1701 (0.0240, 0.4093) | 4.3882 (0.7262, 10.6071) |
| Congo | 0.0503 (0.0144, 0.0935) | 1.2588 (0.4750, 2.2189) |
| Democratic Republic of the Congo | 0.0981 (0.0159, 0.2482) | 2.3777 (0.5454, 5.6427) |
| Equatorial Guinea | 0.0293 (0.0094, 0.0735) | 0.7633 (0.3607, 1.5905) |
| Gabon | 0.0308 (0.0094, 0.0668) | 0.8366 (0.3602, 1.6878) |
| Eastern Sub-Saharan Africa | 0.0740 (0.0157, 0.1436) | 1.8799 (0.5599, 3.4933) |
| Burundi | 0.1234 (0.0138, 0.3137) | 3.0405 (0.5957, 7.3653) |
| Comoros | 0.0588 (0.0124, 0.1315) | 1.4608 (0.4596, 3.0318) |
| Djibouti | 0.0483 (0.0149, 0.1064) | 1.3000 (0.5562, 2.5862) |
| Eritrea | 0.0777 (0.0156, 0.1834) | 2.0471 (0.5272, 4.6128) |
| Ethiopia | 0.0591 (0.0095, 0.1361) | 1.4200 (0.3927, 2.9877) |
| Kenya | 0.0504 (0.0152, 0.0906) | 1.3559 (0.5643, 2.2802) |
| Madagascar | 0.1063 (0.0177, 0.2399) | 2.6266 (0.6771, 5.5066) |
| Malawi | 0.0780 (0.0145, 0.1766) | 1.9719 (0.5282, 4.2121) |
| Mozambique | 0.0960 (0.0166, 0.2109) | 2.5093 (0.6163, 5.2122) |
| Rwanda | 0.0740 (0.0229, 0.1412) | 1.8429 (0.7126, 3.3472) |
| Somalia | 0.2490 (0.0167, 0.7048) | 6.2085 (0.6420, 17.5335) |
| South Sudan | 0.0560 (0.0129, 0.1312) | 1.3883 (0.4810, 2.9469) |
| Uganda | 0.0724 (0.0152, 0.1438) | 1.8003 (0.5161, 3.4645) |
| United Republic of Tanzania | 0.0500 (0.0138, 0.1002) | 1.3035 (0.5122, 2.4299) |
| Zambia | 0.0626 (0.0132, 0.1249) | 1.6386 (0.5059, 3.0701) |
| Southern Sub-Saharan Africa | 0.0942 (0.0643, 0.1166) | 3.0460 (2.3606, 3.7218) |
| Botswana | 0.1122 (0.0602, 0.1995) | 3.4205 (1.9903, 5.4594) |
| Eswatini | 0.1471 (0.0693, 0.2530) | 4.6283 (2.2885, 7.6739) |
| Lesotho | 0.2499 (0.1159, 0.4408) | 7.6495 (3.7887, 12.8666) |
| Namibia | 0.1171 (0.0618, 0.1935) | 3.3430 (2.0259, 5.1305) |
| South Africa | 0.0925 (0.0644, 0.1185) | 3.0738 (2.3872, 3.7969) |
| Zimbabwe | 0.0635 (0.0356, 0.1070) | 1.7873 (1.1949, 2.6491) |
| Western Sub-Saharan Africa | 0.0116 (0.0064, 0.0172) | 0.3792 (0.2262, 0.5750) |
| Benin | 0.0142 (0.0056, 0.0251) | 0.4714 (0.2105, 0.8730) |
| Burkina Faso | 0.0109 (0.0038, 0.0194) | 0.3647 (0.1513, 0.6630) |
| Cabo Verde | 0.0082 (0.0046, 0.0164) | 0.3637 (0.1931, 0.7248) |
| Cameroon | 0.0126 (0.0055, 0.0226) | 0.4431 (0.2034, 0.8338) |
| Chad | 0.0198 (0.0064, 0.0354) | 0.6224 (0.2439, 1.1140) |
| Gambia | 0.0149 (0.0050, 0.0277) | 0.4781 (0.1849, 0.8923) |
| Ghana | 0.0067 (0.0034, 0.0114) | 0.1934 (0.1134, 0.3269) |
| Guinea | 0.0197 (0.0077, 0.0327) | 0.6407 (0.2866, 1.1038) |
| Guinea-Bissau | 0.0235 (0.0081, 0.0445) | 0.7883 (0.2880, 1.5198) |
| Liberia | 0.0094 (0.0030, 0.0189) | 0.3236 (0.1323, 0.6390d) |
| Mali | 0.0235 (0.0090, 0.0390) | 0.7850 (0.3608, 1.3652) |
| Mauritania | 0.0073 (0.0039, 0.0132) | 0.2537 (0.1446, 0.4534) |
| Niger | 0.0200 (0.0046, 0.0378) | 0.6204 (0.1906, 1.1943) |
| Nigeria | 0.0087 (0.0050, 0.0138) | 0.2820 (0.1788, 0.4544) |
| Sao Tome and Principe | 0.0232 (0.0108, 0.0391) | 0.7166 (0.3458, 1.2400) |
| Senegal | 0.0129 (0.0057, 0.0222) | 0.4214 (0.2012, 0.7626) |
| Sierra Leone | 0.0158 (0.0054, 0.0297) | 0.5245 (0.2095, 0.9793) |
| Togo | 0.0153 (0.0060, 0.0268) | 0.5205 (0.2258, 0.9395) |

204 countries are arranged by GBD 7 super regions and 21 regions

ASR: age-standardized rate; DALY: disability-adjusted life years

## Supplementary table 2 Global age- and sex-specific rate for mortality and DALY due to silicosis in 2019

|  | Mortality, per 100,000 | |  | DALY, per 100,000 | |
| --- | --- | --- | --- | --- | --- |
| age group | male | female |  | male | female |
| 15 to 19 | 0.0014 (0.0009, 0.0019) | 0.0006 (0.0004, 0.0009) |  | 0.1039 (0.0687, 0.1423) | 0.0501 (0.0328, 0.0742) |
| 20 to 24 | 0.0032 (0.0022, 0.0047) | 0.0005 (0.0003, 0.0008) |  | 0.2437 (0.1776, 0.3383) | 0.0763 (0.0503, 0.114) |
| 25 to 29 | 0.0072 (0.0053, 0.0101) | 0.0019 (0.0009, 0.0032) |  | 0.5427 (0.4225, 0.7167) | 0.1889 (0.1225, 0.2847) |
| 30 to 34 | 0.0187 (0.0143, 0.0258) | 0.0016 (0.001, 0.0025) |  | 1.6721 (1.2882, 2.1775) | 0.1999 (0.1387, 0.2866) |
| 35 to 39 | 0.0505 (0.0394, 0.0684) | 0.0018 (0.0013, 0.0027) |  | 5.2496 (3.8999, 7.2085) | 0.2556 (0.1796, 0.372) |
| 40 to 44 | 0.1147 (0.0859, 0.1478) | 0.0036 (0.0022, 0.0051) |  | 12.5477 (8.9415, 17.3378) | 0.4191 (0.2936, 0.6045) |
| 45 to 49 | 0.2251 (0.1615, 0.3013) | 0.0057 (0.0038, 0.0083) |  | 24.856 (18.2191, 34.1594) | 0.6388 (0.4542, 0.9181) |
| 50 to 54 | 0.3984 (0.291, 0.5314) | 0.0085 (0.0052, 0.0117) |  | 39.6503 (29.6892, 52.348) | 0.9251 (0.6326, 1.3358) |
| 55 to 59 | 0.5362 (0.4031, 0.6897) | 0.0117 (0.0078, 0.016) |  | 46.4798 (35.0135, 61.0807) | 1.1915 (0.8227, 1.7271) |
| 60 to 64 | 0.7393 (0.5674, 0.9519) | 0.0245 (0.0158, 0.0337) |  | 55.3224 (41.9365, 72.3112) | 1.8291 (1.2776, 2.6589) |
| 65 to 69 | 1.1623 (0.9134, 1.4742) | 0.0321 (0.0204, 0.0439) |  | 68.9558 (53.5621, 88.309) | 2.4986 (1.7535, 3.6768) |
| 70 to 74 | 1.7945 (1.4569, 2.2623) | 0.0649 (0.0451, 0.0891) |  | 77.2352 (61.5908, 96.6216) | 3.4943 (2.4855, 4.8815) |
| 75 to 79 | 3.0625 (2.5425, 4.0204) | 0.1283 (0.09, 0.1717) |  | 88.7259 (72.6381, 110.1041) | 4.5839 (3.3706, 6.1381) |
| 80 to 84 | 5.2039 (4.402, 6.813) | 0.2166 (0.152, 0.2843) |  | 101.5689 (84.8003, 124.042) | 5.533 (4.1171, 7.4352) |
| 85 to 89 | 7.8775 (6.6275, 10.5184) | 0.2725 (0.1918, 0.3773) |  | 103.7295 (88.3735, 126.748) | 5.6684 (4.2248, 7.5622) |
| 90 to 94 | 6.7375 (5.5615, 8.5124) | 0.3204 (0.2226, 0.4486) |  | 66.1431 (55.5219, 79.5998) | 5.3282 (3.9165, 7.1769) |
| 95 plus | 5.289 (4.0422, 6.4536) | 0.4409 (0.2885, 0.6382) |  | 40.0894 (32.9183, 47.443) | 5.1968 (3.8455, 7.0435) |

## Supplementary table 3 Global trend of age-standardized mortality and DALY rates of silicosis by Joinpoint regression, 1990-2019

| **Cohort** | **Segment** | **Lower Endpoint** | **Upper Endpoint** | **APC** | **Lower CI** | **Upper CI** | **Test Statistic (t)** | **Prob > \|t\|** |
| --- | --- | --- | --- | --- | --- | --- | --- | --- |
| Both mortality | 1 | 1990 | 2004 | -2.9* | -3 | -2.9 | -95.5 | < 0.001 |
| Both mortality | 2 | 2004 | 2007 | -3.8* | -5.1 | -2.4 | -5.8 | < 0.001 |
| Both mortality | 3 | 2007 | 2010 | -1.5* | -2.9 | -0.1 | -2.3 | 0.032 |
| Both mortality | 4 | 2010 | 2019 | -3.5* | -3.6 | -3.3 | -58.1 | < 0.001 |
| Female Mortality | 1 | 1990 | 1997 | -1.5* | -1.7 | -1.3 | -20.3 | < 0.001 |
| Female Mortality | 2 | 1997 | 2000 | -0.2 | -1.4 | 1 | -0.4 | 0.705 |
| Female Mortality | 3 | 2000 | 2003 | -1.3* | -2.5 | -0.1 | -2.4 | 0.033 |
| Female Mortality | 4 | 2003 | 2007 | -3.3* | -3.9 | -2.7 | -12 | < 0.001 |
| Female Mortality | 5 | 2007 | 2013 | -1.6* | -1.8 | -1.3 | -12.7 | < 0.001 |
| Female Mortality | 6 | 2013 | 2019 | -1.1* | -1.3 | -0.9 | -11.6 | < 0.001 |
| Male Mortality | 1 | 1990 | 2004 | -3.0* | -3.1 | -2.9 | -91.4 | < 0.001 |
| Male Mortality | 2 | 2004 | 2007 | -3.9* | -5.3 | -2.5 | -5.6 | < 0.001 |
| Male Mortality | 3 | 2007 | 2010 | -1.7* | -3.1 | -0.2 | -2.3 | 0.031 |
| Male Mortality | 4 | 2010 | 2019 | -3.7* | -3.8 | -3.6 | -57.8 | < 0.001 |
| Both DALY | 1 | 1990 | 1994 | -0.3 | -0.8 | 0.2 | -1.4 | 0.195 |
| Both DALY | 2 | 1994 | 2005 | -1.9* | -2 | -1.8 | -34.7 | < 0.001 |
| Both DALY | 3 | 2005 | 2009 | -2.7* | -3.5 | -2 | -7.7 | < 0.001 |
| Both DALY | 4 | 2009 | 2014 | -1.5* | -2 | -1 | -6.6 | < 0.001 |
| Both DALY | 5 | 2014 | 2017 | -4.2* | -5.7 | -2.7 | -5.9 | < 0.001 |
| Both DALY | 6 | 2017 | 2019 | -1.7* | -3.2 | -0.2 | -2.4 | 0.032 |
| Female DALY | 1 | 1990 | 1994 | 2.1* | 1.9 | 2.4 | 19.5 | < 0.001 |
| Female DALY | 2 | 1994 | 2005 | -1.1* | -1.2 | -1.1 | -41.9 | < 0.001 |
| Female DALY | 3 | 2005 | 2010 | -3.5* | -3.7 | -3.3 | -32.8 | < 0.001 |
| Female DALY | 4 | 2010 | 2014 | -0.1 | -0.4 | 0.3 | -0.4 | 0.68 |
| Female DALY | 5 | 2014 | 2017 | -2.1* | -2.8 | -1.4 | -6.2 | < 0.001 |
| Female DALY | 6 | 2017 | 2019 | -0.2 | -1 | 0.5 | -0.7 | 0.489 |
| Male DALY | 1 | 1990 | 1994 | -0.5* | -1 | 0 | -2.3 | 0.04 |
| Male DALY | 2 | 1994 | 2005 | -2.0* | -2.1 | -1.9 | -37.2 | < 0.001 |
| Male DALY | 3 | 2005 | 2009 | -2.7* | -3.4 | -1.9 | -7.7 | < 0.001 |
| Male DALY | 4 | 2009 | 2014 | -1.6* | -2.1 | -1.1 | -7.2 | < 0.001 |
| Male DALY | 5 | 2014 | 2017 | -4.3* | -5.7 | -2.8 | -6.2 | < 0.001 |
| Male DALY | 6 | 2017 | 2019 | -1.7* | -3.2 | -0.2 | -2.5 | 0.027 |

| **Cohort** | **Range** | **Lower Endpoint** | **Upper Endpoint** | **AAPC** | **Lower CI** | **Upper CI** | **Test Statistic~** | **P-Value~** |
| --- | --- | --- | --- | --- | --- | --- | --- | --- |
| Both Mortality | Full Range | 1990 | 2019 | -3.0* | -3.2 | -2.9 | -30.8 | < 0.1 |
| Female Mortality | Full Range | 1990 | 2019 | -1.5* | -1.7 | -1.3 | -15.8 | < 0.1 |
| Male Mortality | Full Range | 1990 | 2019 | -3.2* | -3.4 | -3 | -30 | < 0.1 |
| Both DALY | Full Range | 1990 | 2019 | -2.0* | -2.2 | -1.7 | -17.1 | < 0.1 |
| Female DALY | Full Range | 1990 | 2019 | -1.0* | -1.1 | -0.9 | -18 | < 0.1 |
| Male DALY | Full Range | 1990 | 2019 | -2.1* | -2.3 | -1.8 | -18.2 | < 0.1 |

## Supplementary table 4 Global trend of age-standardized mortality and DALY of silicosis by sex, 1990-2019

| **Year** | **Sex** | **Mortality** | **upper** | **lower** | **DALY** | **upper** | **lower** |
| --- | --- | --- | --- | --- | --- | --- | --- |
| 1990 | Male | 0.8775 | 1.0652 | 0.6368 | 28.8090 | 35.2183 | 22.3066 |
| 1991 | Male | 0.8540 | 1.0306 | 0.6358 | 28.8072 | 34.9277 | 22.7960 |
| 1992 | Male | 0.8287 | 0.9978 | 0.6298 | 28.7151 | 34.7977 | 22.6935 |
| 1993 | Male | 0.8078 | 0.9663 | 0.6206 | 28.5700 | 34.3995 | 23.0103 |
| 1994 | Male | 0.7836 | 0.9281 | 0.6060 | 28.2878 | 34.3522 | 22.6760 |
| 1995 | Male | 0.7657 | 0.9064 | 0.6068 | 27.9579 | 33.8161 | 22.6103 |
| 1996 | Male | 0.7388 | 0.8603 | 0.5892 | 27.3028 | 32.6682 | 22.1793 |
| 1997 | Male | 0.7122 | 0.8244 | 0.5746 | 26.6037 | 32.0219 | 21.4060 |
| 1998 | Male | 0.6923 | 0.7920 | 0.5671 | 25.9808 | 31.2454 | 21.1908 |
| 1999 | Male | 0.6709 | 0.7649 | 0.5504 | 25.3852 | 30.5377 | 20.5196 |
| 2000 | Male | 0.6483 | 0.7374 | 0.5304 | 24.8662 | 30.0805 | 20.3375 |
| 2001 | Male | 0.6268 | 0.7185 | 0.5111 | 24.3753 | 29.4000 | 19.7704 |
| 2002 | Male | 0.6104 | 0.7053 | 0.4987 | 24.0222 | 29.0058 | 19.4810 |
| 2003 | Male | 0.5958 | 0.6825 | 0.4823 | 23.6854 | 28.6914 | 19.2691 |
| 2004 | Male | 0.5737 | 0.6600 | 0.4720 | 23.2099 | 28.2574 | 18.8425 |
| 2005 | Male | 0.5574 | 0.6423 | 0.4581 | 22.7569 | 27.9691 | 18.4333 |
| 2006 | Male | 0.5284 | 0.6087 | 0.4313 | 22.0117 | 26.7823 | 17.6536 |
| 2007 | Male | 0.5105 | 0.5961 | 0.4177 | 21.3547 | 26.1589 | 17.0851 |
| 2008 | Male | 0.5035 | 0.5892 | 0.4145 | 20.8568 | 25.5838 | 16.7078 |
| 2009 | Male | 0.4937 | 0.5794 | 0.4101 | 20.3244 | 24.8639 | 16.5237 |
| 2010 | Male | 0.4845 | 0.5746 | 0.4101 | 19.9565 | 24.5472 | 16.0788 |
| 2011 | Male | 0.4688 | 0.5778 | 0.3950 | 19.6236 | 24.0668 | 15.8707 |
| 2012 | Male | 0.4538 | 0.5459 | 0.3875 | 19.3395 | 23.6241 | 15.6705 |
| 2013 | Male | 0.4348 | 0.5360 | 0.3610 | 18.9592 | 23.4773 | 15.2197 |
| 2014 | Male | 0.4138 | 0.5054 | 0.3471 | 18.5224 | 22.8634 | 14.9121 |
| 2015 | Male | 0.4008 | 0.4979 | 0.3392 | 18.1725 | 22.5741 | 14.6285 |
| 2016 | Male | 0.3882 | 0.4914 | 0.3270 | 17.2733 | 21.4564 | 13.8757 |
| 2017 | Male | 0.3728 | 0.4690 | 0.3151 | 16.3191 | 20.3972 | 13.0469 |
| 2018 | Male | 0.3586 | 0.4589 | 0.3020 | 16.0115 | 19.9500 | 12.7330 |
| 2019 | Male | 0.3480 | 0.4373 | 0.2926 | 15.9073 | 20.1284 | 12.5987 |
| 1990 | Female | 0.0198 | 0.0333 | 0.0143 | 0.8299 | 1.1867 | 0.6163 |
| 1991 | Female | 0.0194 | 0.0327 | 0.0142 | 0.8531 | 1.2186 | 0.6325 |
| 1992 | Female | 0.0191 | 0.0320 | 0.0140 | 0.8719 | 1.2470 | 0.6425 |
| 1993 | Female | 0.0188 | 0.0311 | 0.0138 | 0.8855 | 1.2445 | 0.6625 |
| 1994 | Female | 0.0185 | 0.0305 | 0.0138 | 0.8968 | 1.2548 | 0.6677 |
| 1995 | Female | 0.0183 | 0.0292 | 0.0135 | 0.8995 | 1.2472 | 0.6750 |
| 1996 | Female | 0.0180 | 0.0295 | 0.0136 | 0.8910 | 1.2172 | 0.6627 |
| 1997 | Female | 0.0177 | 0.0284 | 0.0136 | 0.8776 | 1.1866 | 0.6567 |
| 1998 | Female | 0.0176 | 0.0284 | 0.0137 | 0.8651 | 1.1570 | 0.6528 |
| 1999 | Female | 0.0178 | 0.0292 | 0.0138 | 0.8582 | 1.1473 | 0.6500 |
| 2000 | Female | 0.0176 | 0.0284 | 0.0139 | 0.8470 | 1.1283 | 0.6396 |
| 2001 | Female | 0.0173 | 0.0275 | 0.0137 | 0.8392 | 1.1156 | 0.6373 |
| 2002 | Female | 0.0172 | 0.0272 | 0.0138 | 0.8314 | 1.1106 | 0.6358 |
| 2003 | Female | 0.0169 | 0.0267 | 0.0134 | 0.8208 | 1.0852 | 0.6218 |
| 2004 | Female | 0.0164 | 0.0256 | 0.0132 | 0.8088 | 1.0777 | 0.6106 |
| 2005 | Female | 0.0160 | 0.0245 | 0.0128 | 0.7998 | 1.0599 | 0.6055 |
| 2006 | Female | 0.0152 | 0.0230 | 0.0121 | 0.7729 | 1.0236 | 0.5828 |
| 2007 | Female | 0.0148 | 0.0222 | 0.0120 | 0.7441 | 0.9837 | 0.5627 |
| 2008 | Female | 0.0146 | 0.0215 | 0.0118 | 0.7157 | 0.9436 | 0.5484 |
| 2009 | Female | 0.0144 | 0.0205 | 0.0115 | 0.6895 | 0.9099 | 0.5233 |
| 2010 | Female | 0.0141 | 0.0195 | 0.0113 | 0.6748 | 0.8835 | 0.5155 |
| 2011 | Female | 0.0140 | 0.0193 | 0.0110 | 0.6724 | 0.8809 | 0.5129 |
| 2012 | Female | 0.0137 | 0.0183 | 0.0106 | 0.6675 | 0.8753 | 0.5083 |
| 2013 | Female | 0.0135 | 0.0179 | 0.0105 | 0.6645 | 0.8704 | 0.5054 |
| 2014 | Female | 0.0133 | 0.0173 | 0.0101 | 0.6639 | 0.8795 | 0.5008 |
| 2015 | Female | 0.0132 | 0.0170 | 0.0098 | 0.6618 | 0.8703 | 0.5005 |
| 2016 | Female | 0.0130 | 0.0167 | 0.0096 | 0.6422 | 0.8419 | 0.4869 |
| 2017 | Female | 0.0129 | 0.0165 | 0.0096 | 0.6232 | 0.8231 | 0.4742 |
| 2018 | Female | 0.0127 | 0.0164 | 0.0092 | 0.6224 | 0.8247 | 0.4686 |
| 2019 | Female | 0.0126 | 0.0166 | 0.0094 | 0.6243 | 0.8311 | 0.4763 |
